# First Report and Genetic Characterization of Border Disease Virus in Sheep from Hulunbuir, Northeastern China

**DOI:** 10.1155/2024/9924724

**Published:** 2024-01-31

**Authors:** Yongxu Yuan, Liang Li, Ziyan Liu, Xing Yang, Wei Wang, Wenbo Xu, Ning Liu, Liyan Sui, Yinghua Zhao, Quan Liu, Zedong Wang

**Affiliations:** ^1^Changchun Veterinary Research Institute, Chinese Academy of Agricultural Sciences, Changchun, Jilin, China; ^2^College of Food Science and Engineering, Tonghua Normal University, Tonghua, Jilin, China; ^3^Department of Infectious Diseases, Center of Infectious Diseases and Pathogen Biology, Key Laboratory of Organ Regeneration and Transplantation of the Ministry of Education, State Key Laboratory of Zoonotic Diseases, The First Hospital of Jilin University, Changchun, Jilin, China; ^4^Department of Medical Microbiology and Immunology, School of Basic Medicine, Dali University, Dali, Yunnan, China; ^5^Hulunbuir Animal Disease Control Center, Inner Mongolia Autonomous Region, Hailar, China

## Abstract

Border disease virus (BDV), a member of the *Pestivirus* genus within the *Flaviviridae* family, is known to inflict significant economic losses on livestock farms due to its association with reproductive disorders and persistent infections in sheep and goats. However, comprehensive epidemiological investigations of BDV in China are scarce. This study examined BDV infection in sheep from Hulunbuir, Inner Mongolia, northeastern China, utilizing metagenomic sequencing and polymerase chain reaction (PCR) assay. Among the 96 serum samples analyzed, only one tested positive for BDV nucleotide sequence, yielding a prevalence rate of 1.0%. A total of 11,985 nt long genome sequences was amplified, which showed nucleotide identities ranging from 76.6% to 87.2% and amino acid identities ranging from 85.2% to 93.2% with other BDV strains worldwide. Phylogenetic analysis unequivocally placed the viral strain within genotype BDV-3, showing a close genetic affinity with strain JSLS12-01 identified in Jiangsu province, China. Furthermore, selection pressure analyses suggested that purifying selection predominantly influenced the evolutionary dynamics of BDV genomes. This study marks the inaugural detection of BDV in sheep within Inner Mongolia, northeastern China, thereby enhancing our understanding of the extensive genetic diversity and geographical distribution of BDV strains across the country. These findings hold relevance for the livestock industry and disease surveillance efforts, offering valuable insights into the prevalence and genetic characteristics of BDV in this region.

## 1. Introduction

Border disease virus (BDV), a member of the *Pestivirus* genus within the *Flaviviridae* family, is primarily recognized for its infection in small ruminants, particularly sheep and goats, leading to the emergence of border disease across various regions [1]. In addition to BDV, the *Pestivirus* genus encompasses notable species: classical swine fever virus (CSFV), bovine viral diarrhea virus type 1 (BVDV-1), and BVDV-2. These viruses share key features as single-stranded positive-sense RNA viruses, characterized by a singular open reading frame encoding a polyprotein, subsequently cleaved by both viral and cellular proteases into N^pro^, capsid (*C*), three envelope proteins (E^rns^, E1, and E2), and seven nonstructural proteins (p7, NS2, NS3, NS4A, NS4B, NS5A, and NS5B) [1, 2].

**Figure 1:**
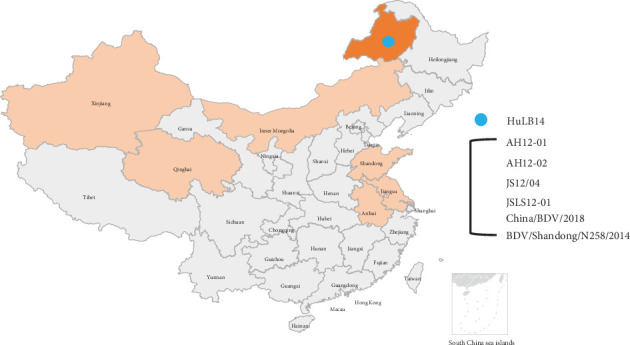
Border disease virus (BDV) strains identified in China. The orange areas on the map represent provinces in China where BDV has been positively identified. The blue circle indicates the site where the samples were collected.

Since its initial identification in lambs from the border region of England and Wales in 1959 [3], BDV has been detected in numerous countries, including Turkey, Germany, Italy, USA, Australia, India, Japan, and China [4]. The virus has been linked to various health issues in sheep and goats. Infected animals often present reproductive disorders, with neonatal lambs particularly vulnerable to infertility, abortion, congenital tremors, skeletal anomalies, and the birth of debilitated “hairy shaker” lambs [5, 6]. In China, the initial documentation of BDV was in goat herds exhibiting diarrhea in the Anhui and Jiangsu provinces in the eastern part of the country [7]. Subsequently, the virus was identified in sheep populations in Jiangsu and Shandong provinces and Tibetan sheep residing in Qinghai province [1, 8, 9]. A significant occurrence arose in 2019 when BDV was detected in *Melophagus ovinus* (sheep ked) collected from sheep in Xinjiang, China [24]. BDV strains are categorized into eight genotypes (BDV-1–BDV-8) based on 5′-UTR and N^pro^ regions of the virus, with all Chinese isolates belonging to BDV-3 (Figure 1).

Hulunbuir, situated in Inner Mongolia, northeastern China (47°05″–53°20″N, 115°31″–126°04″E), is renowned for sheep and cattle rearing due to its rich herbage and forestry resources [10]. Proactive disease surveillance, prevention, and management remain paramount to mitigate economic losses in the local animal husbandry sector. In this study, we employed metagenomic analysis to identify the BDV infection among sheep in Hulunbuir. Whole genome analysis of the virus confirmed its classification within genotype BDV-3. These findings provide valuable insights for enhancing the region's disease surveillance and control strategies.

## 2. Materials and Methods

### 2.1. Sample Collection

From April to July 2021, blood samples were meticulously collected from free-ranging sheep in Hulunbuir, situated in the Inner Mongolia Autonomous Region, northeastern China. The serum samples were separated through centrifugation at 500 rpm for 10 min and stored at −80°C until use.

### 2.2. Metagenomic Sequencing and Analyses

Viral metagenomic sequencing was conducted as previously described [11]. Briefly, the serum samples were digested with micrococcal nuclease (NEB, USA) at 37°C for 2 hr and pooled for RNA extraction using a TIANamp Virus RNA Kit (TIANGEN, China). Metagenomic sequencing was performed at Tanpu Biological Technology (Shanghai, China) using an Illumina NovaSeq 6000 System.

The sequenced raw data were analyzed as previously described [12]. First, the BBMap program (https://github.com/BioInfoTools/bbmap) was used to remove low-quality reads, ribosomal RNA, host contamination, and bacteria sequences. The reads were then assembled into contigs via SPAdes v3.14.1 (https://github.com/ablab/spades) and SOAPdenovo v2.04 (https://github.com/aquaskyline/SOAPdenovo-Trans) [13, 14]. Host and bacterial sequences were removed after mapping against the sequences in the nonredundant nucleotide (nt) and protein (nr) databases downloaded from GenBank with BLAST + v2.10.0. The viruses were identified by mapping the reads back to the assembled contigs in Bowtie2 v2.3.3.1.

### 2.3. BDV Detection and Genome Amplification

RNA was extracted from the serum samples using the TIANamp Virus RNA Kit (TIANGEN, China) and converted to complementary DNA using a Reverse Transcription kit (TaKaRa, Japan). BDV was detected using seminested reverse transcription-polymerase chain reaction (RT-PCR) with primers designed based on the sequenced contigs, F1 (5′-GACTACCATTACGACCTCCT-3′) and R (5′-GCCTGATATCCAACGTACC-3′) for the first round, and F2 (5′-TTGGCTTACAACAGCTACGAA-3′) and R for the second round. To obtain the complete genome of BDV-positive samples, specific primers were designed based on the sequenced BDV contigs and genome sequences of BDV strains downloaded from the GenBank (Table S1) using Vector NTI V3.0 software. PCR products were recovered using a gel extraction kit (TIANGEN, China) and sequenced.

### 2.4. Genome Characterization and Phylogenetic Analyses

ORFfinder, available in NCBI (https://www.ncbi.nlm.nih.gov/orffinder), was used to predict the virus's potential open reading frames (ORFs). N-Glycosylation sites were analyzed using NetNGlyc1.0, available from http://www.cbs.dtu.dk/services/NetNGlyc. Sequence identities were assessed using the MegAlign program available within the DNAstar package V7.0. Moreover, the aligned BDV amino acid sequences were conducted for similarity plot and bootscan analysis using Simplot version 3.5.1 [15]. Phylogenetic trees were generated based on the nucleotide sequences of the polyprotein-coding regions and 5′-UTR of BDV strains, using the maximum likelihood (ML) method available within MEGA version 7.0 software [16] with a bootstrap of 1,000 replicates. The bootstrap values ≥70 were considered significant and displayed in the trees.

### 2.5. Selection Pressure Analyses

To evaluate and compare the selection pressure involved in the evolution of the pestiviruses genome [16, 17], two major species, including BVDV-1 and CSFV (Table S3), were used together with BDV to calculate the numbers of synonymous nucleotide substitutions per synonymous site (dS) and nonsynonymous substitutions per nonsynonymous site (dN) using the Nei–Gojobori model available from MEGA 7.0. The site-specific selection pressures were evaluated using the single-likelihood ancestor counting (SLAC) method supported by the Datamonkey web server (http://www.datamonkey.org), and the difference between the dN and dS rates (dN−dS) of each codon was plotted.

To evaluate the putative positive selection sites across the polyprotein-coding region of the three viral species, four methods, including SLAC, fast unconstrained Bayesian approximation (FUBAR), fixed effects likelihood (FEL), and mixed effects model of evolution (MEME) available within the Datamonkey web were used. Codons with *p*  < 0.05 or a posterior probability >0.95 identified by at least three methods were considered positive selections [18, 19].

## 3. Results

### 3.1. Sample Collection and BDV Identification

A comprehensive collection of 96 blood samples was procured from sheep located in Hulunbuir, situated in northeastern China (Figure 1). We obtained two BDV sequences, each approximately 200 nt in length, using metagenome sequencing. These sequences exhibited nucleotide identities ranging from 88.6% to 91.5% compared to the BDV strain JSLS12-01, previously isolated from sheep in Jiangsu province, China. The seminested RT-PCR technique facilitated the detection of BDV presence in only one of the serum samples. We obtained a viral genome spanning 11,985 base pairs, designated as HuLB14 (with GenBank accession number: OQ378958).

### 3.2. Genome Characterization

**Figure 2:**
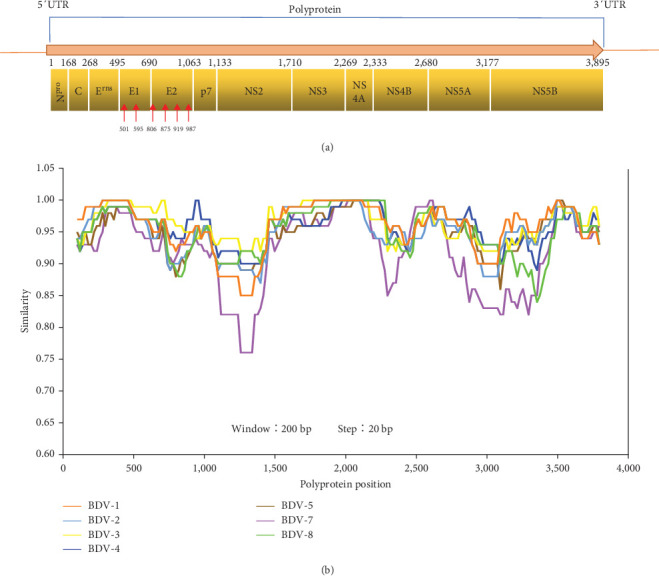
Genomic characterization of border disease virus (BDV). (a) Genome organization of BDV. N-Linked glycosylation sites are indicated with red arrows. (b) Simplot analysis of BDV strains of subtype 1–8 based on polyprotein. Comparison of nucleotide sequence identity of polyprotein within the subtype 1–8 of BDV listed in Table S3.

The HuLB14 strain encoded a polyprotein spanning 3,897 amino acids (aa). This precursor undergoes subsequent cleavage, orchestrated by viral and cellular proteases, yielding five structural proteins and seven nonstructural proteins (N^pro^-C-E^rns^-E1-E2-p7-NS2-NS3-NS4A-NS4B-NS5A-NS5B; Figure 2(a)). Notably, the cleavage sites responsible for polyprotein processing demonstrated remarkable conservation across various BDV strains (Table 1). Two and four N-glycosylation sites were identified within the putative E1 and E2 proteins (Figure 2(a)). Employing SimPlot analysis, a distinctive amino acid similarity trend emerged among the seven BDV genotypes (excluding BDV-6 strains lacking complete genomes) in p7, NS2, NS4B, NS5A, and NS5B proteins. Notably, the BDV strain discovered in this study exhibited the lowest amino acid similarity of 75% with BDV-7 strains, indicating divergence in these specific protein regions (Figure 2(b)).

### 3.3. Sequence Comparison and Phylogenetic Analyses

**Figure 3:**
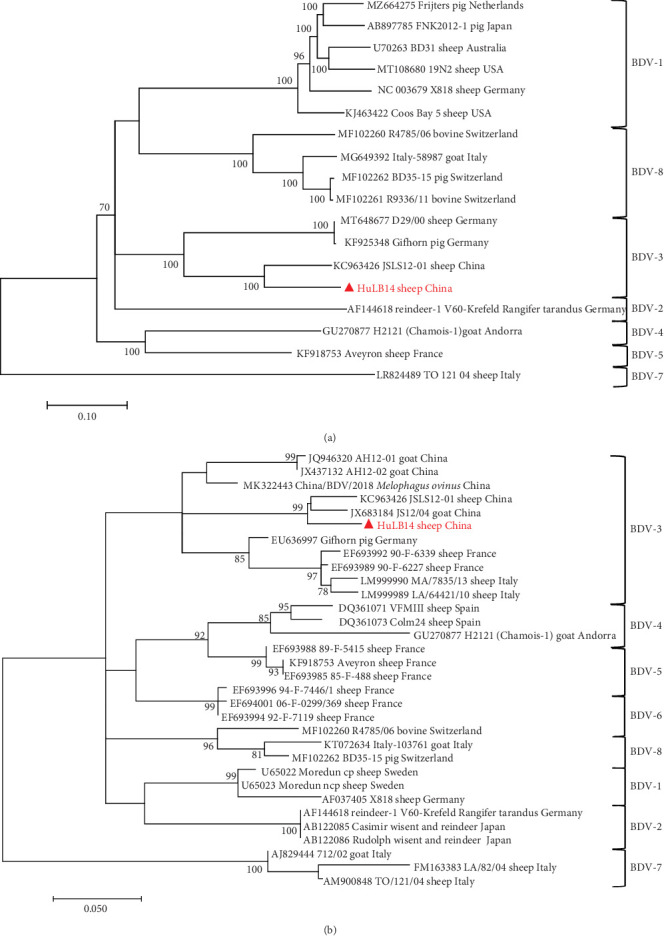
(a) Phylogenetic tree of nucleotide sequences based on the polyprotein of border disease virus (BDV). (b) Phylogenetic tree of nucleotide sequences based on the 5′-UTR of BDV. A bootstrapping analysis of (a) 1,000 and (b) 10,000 replicates was conducted, respectively, and the bootstrap values of more than 70 were shown in the trees. The red triangle indicates the identified BDV strain.

The HuLB14 strain exhibited a notably higher sequence identity with genotype BDV-3 strains compared to other genotypes, which was evident in nucleotide and amino acid comparisons, with identities ranging from 80.1% to 87.2% for nucleotides and 89.3% to 93.2% for amino acids (Table S2). Furthermore, the HuLB14 strain displayed substantial similarity, sharing 76.6%–86.6% nucleotide identity and 82.2%–86.7% amino acid identity with BDV strains from various genotypes worldwide (Table S2). Phylogenetic analyses provided insights into the relationships among BDV strains, revealing their classification into eight distinct phylogenetic groups (BDV-1–BDV-8). Through these analyses, the HuLB14 strain was observed to cluster closely with BDV-3 strains identified in different regions, including China, Germany, France, and Italy (Figure 3).

### 3.4. Selection Pressure of BDV Genome

**Figure 4:**
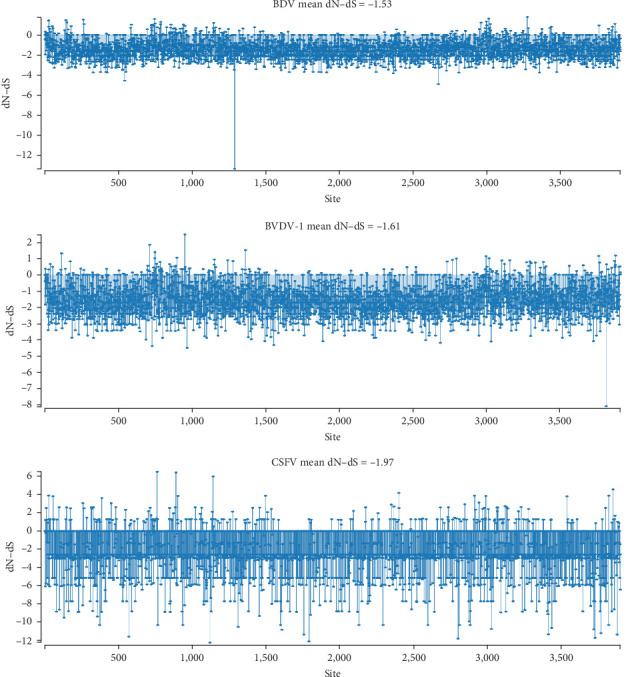
Differences between nonsynonymous and synonymous (dN−dS) rates across the polyprotein-coding region of border disease virus (BDV), bovine viral diarrhea virus type 1 (BVDV-1), and classical swine fever virus (CSFV). The rates dN−dS > 0 indicate a positively selected site, while dN−dS < 0 indicates a negatively selected site.

The genome sequences of BDV, BVDV-1, and CSFV strains were analyzed to determine dN and dS values for each site. This analysis revealed a predominance of negatively selected sites (dN−dS < 0) throughout the polyprotein-coding region of pestiviruses (Figure 4). The mean dN−dS values for BDV, BVDV-1, and CSFV strains were −1.53, −1.61, and −1.97, respectively. Notably, dN/dS ratios for the coding sequences of the three pestiviruses' 12 proteins were consistently below 1, except for the E2 protein of BVDV-1 (Table 2). Intriguingly, prediction using three out of the four methods (SLAC, FEL, FUBAR, and MEME) identified 1, 1, and 0 putative sites of positive selection in the polyprotein-coding region of BDV, BVDV-1, and CSFV, respectively (Table 3).

## 4. Discussion

BDV has been confirmed in various eastern and northwestern China provinces, including Shandong, Jiangsu, Anhui, Xinjiang, and Qinghai. However, this study marks the first identification of BDV in Inner Mongolia, located in northeastern China. These findings significantly broaden the geographical scope of BDV infections within China. Notably, the sheep found positive for BDV in this study were 6 months old and exhibited no discernible clinical symptoms. This lack of symptoms could be attributed to the relatively weak pathogenicity associated with the BDV-3 genotype. A relevant comparison can be drawn from the virus strain JSLS12-01, discovered in Jiangsu, which shares a close genetic relationship with the identified virus. JSLS12-01 induces only mild clinical symptoms, such as temporary depression and a brief fever lasting around 5 days, particularly in 1-month-old sheep [1]. The earlier study involving JSLS12-01 also revealed that a considerable portion of the sheep population (44.4%) showed serological positivity for BDV, indicating prior exposure to the virus. Notably, only one sheep from the group demonstrated slow growth, possibly linked to persistent viral infection [1].

Pestiviruses, encompassing BVDV, CSFV, and BDV, have demonstrated the capacity to infect various animal species, particularly domestic and wild artiodactyla. These instances of cross-species infections have been documented in various studies [20, 21]. While sheep is the natural host species for BDV, research unequivocally demonstrates that BDV is capable of infecting other animals, including goats [7], cattle [22], pigs [2], and wild boars [23]. In this study, although cattle and sheep typically share the same pasture in Hulunbuir, our cattle herd samples yielded no positive results (data not shown).

Furthermore, we analyzed the prevalence of purifying selection as the primary evolutionary force shaping the genomes of pestiviruses, employing dN/dS ratio analyses and site-specific selection pressure assessments. Notably, the polyprotein-coding region of BDV and BVDV-1 contains one and two putative positively selected codons, respectively, as predicted by at least three of the four methods (SLAC, FEL, FUBAR, and MEME). However, further investigations are necessary to elucidate the functional implications of these potentially positively selected sites.

This study did not include serological tests for BDV, which may lead to an underestimation of BDV infection prevalence. Since sheep infected with BDV experience a brief period of viremia, serological detection could be suitable for epidemiological monitoring of BDV [1]. Nevertheless, the serological cross-reactions induced by pestiviruses should be rigorously substantiated [2, 22]. The source of the virus remains unclear, and it is worth noting that sheep, especially lambs, are frequently transported across provinces in China, which could facilitate BDV transmission and contribute to the virus's geographical spread. Active surveillance, prevention, and control measures are crucial for managing border diseases and mitigating economic losses.

In conclusion, this study represents the first detection of BDV in sheep from Inner Mongolia, northeastern China, significantly enhancing our understanding of the extensive genetic diversity and geographical distribution of BDV in the country. Further research is warranted to investigate the virulence and epidemiological distribution of BDV.

## Figures and Tables

**Table 1:** Comparison of predicted border disease virus (BDV) polyprotein cleavage sites.

Virus	Cleavage site at									
	N^pro^/C	C/E^rns^	E^rns^/E1	E1/E2	E2/P7	P7/NS2	NS2/NS3	NS3/NS4A	NS4A/NS4B	NS4A/NS5A
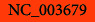	TSC/SDD	VAP/ENV	ANA/QSP	AQG/QFA	TSA/INL	VKG/EGA	ILR/GPA	TGL/SAA	KEL/AQG	RRL/SGN
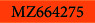	…/…	.TS/…	…/…	…/…	…/MT.	…/D..	…/…	…/…	…/…	.S./…
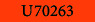	…/…	.KP/..I	…/…	.K./E..	…/MS.	…/D..	…/…	…/.T.	…/…	.S./…
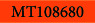	…/…	.TP/..I	.Y./…	…/…	…/MS.	…/D..	…/…	…/…	…/…	.N./…
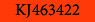	…/…	…/..I	…/…	…/…	A../MC.	…/D.T	…/…	…/…	…/…	. S./…
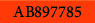	…/…	…/..I	…/…	…/…	…/MS.	…/…	…/…	…/.T.	…/…	.S./…
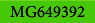	…/…	A.A/..I	…/…	…/…	A../MG.	…/.ET	…/…	…/…	…/…	.N./…
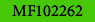	…/…	.SM/..I	…/…	…/…	A../MG.	…/.ET	…/…	…/.T.	…/…	.N./…
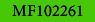	…/…	.SM/..I	…/…	…/…	A../MG.	…/.ET	…/…	…/.T.	…/…	.N./…
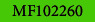	…/…	.S./..I	…/…	…/…	A../KGF	.R./.ET	…/…	A../…	…/…	.N./…
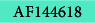	…/…	.LS/..I	…/…	.N./..S	AA./MS.	.R./.ET	…/…	…/…	…/…	.N./…
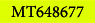	…/…	.TS/..I	…/…	…/…	A../VS.	…/SDV	…/…	A../…	…/…	.N./…
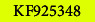	…/…	.TS/..I	…/…	…/…	A../VS.	…/SDV	…/…	A../…	…/…	.N./…
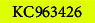	…/…	.TS/..I	…/…	…/..T	A../SG.	…/SEV	…/…	S../.T.	…/…	.N./…
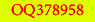	A../…	.TS/..I	…/…	…/.L.	A../LS.	…/SEV	…/…	…/…	…/…	.N./…
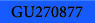	…/…	.T./..I	…/…	…/…	.T./MS.	.R./AEV	…/…	…/…	…/…	.N./…
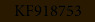	…/…	TSS/..I	…/…	…/…	AN./.S.	…/AET	…/…	…/…	…/…	.N./…
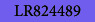	…/..E	.TS/..I	…/.T.	…/..T	AA./.S.	…/.QE	…/…	A../…	…/…	.T./.S.

Slashes (/) indicate cleavage. The BDV strains marked with different colors indicate different genotypes of BDV. BDV-1 (Orange), BDV-2 (Cyan), BDV-3 (Yellow), BDV-4 (Blue), BDV-5 (Brown), BDV-7 (Purple), BDV-8 (Green). BDV-6 was not listed in the table due to the need for a complete genome. OQ378958 marked with red color was the BDV strain identified in this study.

**Table 2:** The mean numbers and ratios of nonsynonymous (dN) and synonymous (dS) substitutions in different genes of border disease virus (BDV), bovine viral diarrhea virus type 1 (BVDV-1), and classical swine fever virus (CSFV).

Gene	BDV (*n* = 18)			BVDV−1 (*n* = 27)			CSFV (*n* = 18)		
	dN	dS	dN/dS	dN	dS	dN/dS	dN	dS	dN/dS
N^pro^	0.128	1.397	0.0916	0.083	1.311	0.0633	0.028	0.31	0.09
C	0.08	1.092	0.0733	0.063	1.669	0.0377	0.018	0.484	0.037
E^rns^	0.034	1.217	0.0279	0.046	1.911	0.0241	0.03	0.437	0.069
E1	0.062	1.615	0.0384	0.129	0.93	0.1387	0.016	0.517	0.031
E2	0.157	1.637	0.0959	0.302	0.219	1.379	0.032	0.399	0.08
P7	0.082	1.626	0.0504	0.129	1.443	0.0894	0.033	0.824	0.04
NS2	0.088	1.932	0.0455	0.188	0.49	0.3837	0.025	0.564	0.044
NS3	0.015	1.79	0.0084	0.015	1.437	0.0104	0.006	0.428	0.014
NS4A	0.023	1.409	0.0163	0.019	1.387	0.0137	0.003	0.326	0.009
NS4B	0.046	1.76	0.0261	0.131	0.505	0.2594	0.019	0.403	0.047
NS5A	0.137	1.854	0.0739	0.109	1.55	0.0703	0.03	0.41	0.073
NS5B	0.08	1.944	0.0412	0.1	0.809	0.1236	0.018	0.471	0.038

**Table 3:** Prediction of putative positive selection site of BDV genome from different hosts (*p* < 0.05 or posterior probability > 0.95).

Model	BDV (*n* = 18)	BVDV-1 (*n* = 27)	CSFV (*n* = 18)
SLAC	**3274**	**114 953**	**0**

FEL	28 150 740 787 2959 3012 **3274**	**114 953** 1364 2798	**761** 889

FUBAR	0	**953**	**761**

MEME	28 146 159 718 725 735 739 744 745 747 748 771 776 779 787 813 834 850 852 853 855 860 866 883 955 975 1025 1026 1186 1195 2411 2743 2753 2763 2800 2806 2838 2899 2918 2959 3012 3023 3201 3213 3214 3216 3217 3219 3221 **3274** 3828	21 **114** 181 296 392 421 488 606 607 609 613 671 706 716 724 729 733 740 741 749 772 778 785 842 850 872 875 883 905 952 **953** 955 962 970 1005 1034 1098 1138 1244 1297 1364 1368 1425 1697 1894 2269 2496 2800 2898 2987 2995 3017 3026 3135 3192 3280 3291 3338 3401 3443 3473 3593 3715 3717 3721 3753 3758 3762 3771 3793 3794 3806 3812 3819 3828	11 173 316 757 889 1140 1496 2363 2988 3087 3887

Locations determined to be positive selections by at least three methods are shown in bold. The number of GenBank logins for the viruses used in this analysis was similar to Table S3.

## Data Availability

The data are available upon reasonable request. Additional supporting information may be found in the online version of the article at the publisher's website.

## Supplementary Materials

Supplementary Materials Table S1: the primers used for the genome amplification of border disease virus (BDV). Table S2: the sequence identities of the border disease virus (BDV) polyprotein gene at the nucleotide (upper right) and amino acid (lower left) levels. Table S3: the study used the viral strains for phylogenetic and selection pressure analyses.
