# Supplementary material for: First Report and Genetic Characterization of Border Disease Virus in Sheep from Hulunbuir, Northeastern China
Source: Transbound Emerg Dis. 2024 Jan 31;2024:9924724. doi: 10.1155/2024/9924724 (PMC12017182; doi:10.1155/2024/9924724)
Supplement: Supplementary Materials — Table S1: the primers used for the genome amplification of border disease virus (BDV). Table S2: the sequence identities of the border disease virus (BDV) polyprotein gene at the nucleotide (upper right) and amino acid (lower left) levels. Table S3: the study used the viral strains for phylogenetic and selection pressure analyses. [file 9924724.f1.docx]

Supplement files

TABLE S1. The primers used for the genome amplification of border disease virus (BDV).

| Primer | Position (bp)^*^ | Sequence (5'→3') | Polarity | Amplicon (bp) |
| --- | --- | --- | --- | --- |
| Detection |  |  |  |  |
| BDV F | 6616 | GACTACCATTACGACCTCCT | + | 222 |
| BDV R2 | 6838 | TTGGCTTACAACAGCTACGAA | - |  |
| BDV R1 | 7124 | GCCTGATATCCAACGTACC | - |  |
| Genome amplification |  |  |  |  |
| 1 BDV F | 6 | GCCCTTAGTAGGACTAGCAAA | + | 501 |
| 1 BDV R2 | 507 | TGATGTATATGCCACTTACAGG | - |  |
| 1 BDV R1 | 548 | ACGGGTTTCACATAATCCTGA | - |  |
| 2 BDV F | 260 | TCTCTGCTGTACATGGCAC | + | 577 |
| 2 BDV R2 | 694 | TACTCGCCAATCTAAACCGTA | - |  |
| 2 BDV R1 | 837 | TGATCTTCATAGCTCCTCG | - |  |
| 3 BDV F | 511 | TGAGTGGCATATACATCAAGC | + | 524 |
| 3 BDV R2 | 1035 | CTATGATAGCCCAAGCCAG | - |  |
| 3 BDV R1 | 1072 | AGTTATATTCTCTGAAGTCACGG | - |  |
| 4 BDV F | 728 | CTCCGATGACAACAAGAGCA | + | 441 |
| 4 BDV R2 | 1223 | TCCCCTTTAGTTCTGCGTCT | - |  |
| 4 BDV R1 | 1272 | AGCCTACAACATGTATAATTGGT | - |  |
| 5 BDV F | 1367 | TAAAACCCAGGCTAATCTGACC | + | 471 |
| 5 BDV R2 | 1766 | GTCACATTACAATAGGGCGAT | - |  |
| 5 BDV R1 | 1838 | TACTATCTTTGTGTTCCCTGGC | - |  |
| 6 BDV F | 1698 | AAACAGTTAGGCATAATGGG | + | 385 |
| 6 BDV R2 | 2083 | GGACGGTATCACATCCTCCA | - |  |
| 6 BDV R1 | 2205 | TCTTATAGCTCTTAGGGCCAA | - |  |
| 7 BDV F1 | 2050 | CGAGCCAACTGAACTTGACA | + | 209 |
| 7 BDV F2 | 2259 | ACAGCTTTTCTGATATGCCTA | + |  |
| 7 BDV R | 2607 | TTCCTCTGTAACCATGGCTGA | - |  |
| 8 BDV F1 | 2259 | ACAGCTTTTCTGATATGCCTA | + | 490 |
| 8 BDV F2 | 2298 | CAAATAGTGCAAGGACTAATCT | + |  |
| 8 BDV R | 2788 | TTCACCACTTCAACGGCTA | - |  |
| 9 BDV F | 2809 | TATACAGGAGGAGCAAGCCAT | + | 547 |
| 9 BDV R2 | 3356 | TATACAGGAGGAGCAAGCCAT | - |  |
| 9 BDV R1 | 3384 | ACCTAGTAGAGCCACTACAGC | - |  |
| 10 BDV F | 3510 | ATAACACACAGAGATCCCGAG | + | 323 |
| 10 BDV R2 | 3833 | TATAGCAAGGTCTACCCCAGGA | - |  |
| 10 BDV R1 | 4080 | CAGAACTCCTGCAATATCCAA | - |  |
| 11 BDV F | 3708 | TTCGACCTGCAGTTCACCT | + | 526 |
| 11 BDV R2 | 4234 | GCTGACTTTGCCTATCCAGT | - |  |
| 11 BDV R1 | 4288 | ATATACCCCTTCTTGTGTCT | - |  |
| 12 BDV F | 4387 | TAAGTAGCAAATGGCAGTGT | + | 497 |
| 12 BDV R2 | 4884 | TAGCCCTTGTACTCTTCTCTG | - |  |
| 12 BDV R1 | 4963 | CACTAGGAGCAGTTTTACCTT | - |  |
| 13 BDV F | 4579 | CAAGTAGGATAAAGAGCCTGA | + | 415 |
| 13 BDV R2 | 4994 | AGATCCCCTACTTCTGAGC | - |  |
| 13 BDV R1 | 5065 | ACATGGCATCTCTCATGGTC | - |  |
| 14 BDV F | 5150 | GATCAGATTCCCCACGTCA | + | 466 |
| 14 BDV R2 | 5616 | GAYACCGTYTGTATTCCACT | - |  |
| 14 BDV R1 | 5689 | TRATTTGCTTRAACTCCCCTC | - |  |
| 15 BDV F | 5224 | GAATCAGTTCGGTTGACCAT | + | 344 |
| 15 BDV R2 | 5568 | GTTCTTCCCGACTTTTACCCT | - |  |
| 15 BDV R1 | 5617 | TAGACACCGTTTGTATTCCAC | - |  |
| 16 BDV F | 5410 | TATCAGGAACTAAAGGAGCCAT | + | 357 |
| 16 BDV R2 | 5767 | ACACTCTTTTRTGCCTRCCAA | - |  |
| 16 BDV R1 | 5822 | YTGCCTCATRTAYTGRTACACA | - |  |
| 17 BDV F | 5730 | AAGACAACTGAGCTGCCTA | + | 491 |
| 17 BDV R2 | 6221 | CCAGCATRTTRCCTTTCATCTCY | - |  |
| 17 BDV R1 | 6298 | AGTAACCTGAATTATACCCYT | - |  |
| 18 BDV F1 | 6028 | TGGCTATAATAGGAAAGATCCAC | + | 499 |
| 18 BDV F2 | 6136 | AGGAATTCATAGCTCCAGA | + |  |
| 18 BDV R | 6635 | TRCCATACCTTTGAGCYTGT | - |  |
| 19 BDV F | 6579 | TACTACAGGAGCCAGGAAACA | + | 514 |
| 19 BDV R2 | 7093 | CCACYARAAGAGCATTCTCG | - |  |
| 19 BDV R1 | 7120 | CCTGATATCCAACGTACCCAA | - |  |
| 20 BDV F | 7152 | AGACACGTACCYATAGTCACA | + | 535 |
| 20 BDV R2 | 7685 | CTAGTAAACTTGCCACGAAC | - |  |
| 20 BDV R1 | 7753 | GCAGGCTCTACTATCTTAGACA | - |  |
| 21 BDV F1 | 7573 | GGTGARTCAGTAAAYGACCA | + | 560 |
| 21 BDV F2 | 7627 | TACATCATCAACAGACCCCAA | + |  |
| 21 BDV R | 8187 | CAGCCTTTGTAGAACACTCC | - |  |
| 22 BDV F1 | 8009 | TGCMATAGAATCAAGTGAGC | + | 458 |
| 22 BDV F2 | 8065 | ATTTCTTAGAYCAGGCAGCCA | + |  |
| 22 BDV R | 8523 | CAGTCTCAATTCTCCTGCACA | - |  |
| 23 BDV F | 8037 | ACGCTACTGATGAAAGTGTT | + | 486 |
| 23 BDV R2 | 8523 | TGTGCAGGAGAATTGAGACTG | - |  |
| 23 BDV R1 | 8625 | TCTAATCTCCTCAAGGTCGTC | - |  |
| 24 BDV F1 | 8986 | AGAGAAATCCCCACAGCAA | + | 429 |
| 24 BDV F2 | 9043 | GATTTAGGCACGATCAAACCC | + |  |
| 24 BDV R | 9472 | GCAGGTCTTTCTCTATATTGGC | - |  |
| 25 BDV F | 9404 | ATATAAAGGAAACAACGCAAC | + | 340 |
| 25 BDV R2 | 9744 | TTGGGAGCYACYGAYTCCA | - |  |
| 25 BDV R1 | 9919 | TGCCAACTGATATGCTGACA | - |  |
| 26 BDV F | 10233 | ACAATGTCTTCCGTAGGGAT | + | 504 |
| 26 BDV R2 | 10724 | CTACCGGCTTTTGTTTGACC | - |  |
| 26 BDV R1 | 10857 | TTTACTGGTTACCTGCGTGTC | - |  |
| 27 BDV F | 10692 | AGGTTGGCAATAACAAAGGT | + | 491 |
| 27 BDV R2 | 11133 | ATCCCCACAGACATGTATCTTG | - |  |
| 27 BDV R1 | 11184 | CTGTTGGCAAATTTCTCTCCC | - |  |
| 28 BDV F | 11067 | CTTGACTATGATCTACGCCTT | + | 441 |
| 28 BDV R2 | 11571 | ACCTCTTTATATGCAGCGATT | - |  |
| 28 BDV R1 | 11631 | AGTTTAATAATGCCAGTTTCTCA | - |  |
| 29 BDV F1 | 11376 | CCTAGCTAAAATGGCTACGAGA | + | 395 |
| 29 BDV F2 | 11521 | CCGTGTATCAACCCAAGCA | + |  |
| 29 BDV R | 11916 | AAGACCTTAAGCCTTCTGAGT | - |  |
| 30 BDV F1 | 11376 | CCTAGCTAAAATGGCTACGAGA | + | 440 |
| 30 BDV F2 | 11521 | CCGTGTATCAACCCAAGCA | + |  |
| 30 BDV R | 11961 | AGCATCTTTTAAATCCGAACC | - |  |

TABLE S2. The sequence identities of border disease virus (BDV) polyprotein gene at the nucleotide (upper right) and amino acid (lower left) levels calculated using MegAlign program available within DNAstar V7.1. The compared strains are represented with GenBank accession number and discovered country, and different colors indicate different genotypes of BDV strains. BDV-1 (Orange), BDV-2 (Cyan), BDV-3 (Yellow), BDV-4 (Blue), BDV-5 (Brown), BDV-7 (Purple), BDV-8 (Green).

| **Strains** | 1 | 2 | 3 | 4 | 5 | 6 | 7 | 8 | 9 | 10 | 11 | 12 | 13 | 14 | 15 | 16 | 17 | 18 |
| --- | --- | --- | --- | --- | --- | --- | --- | --- | --- | --- | --- | --- | --- | --- | --- | --- | --- | --- |
| 1 NC_003679/Germany |  | 89.8 | 89.2 | 88.7 | 89.5 | 89.1 | 77.5 | 77.5 | 77.4 | 77.4 | 76.7 | 77 | 77 | 77 | 76.7 | 76.4 | 76.9 | 73 |
| 2 MZ664275/Netherlands | 94 |  | 90.6 | 90.4 | 91.8 | 89.5 | 77.2 | 77.3 | 77.2 | 77.7 | 76.7 | 77.3 | 77.2 | 77.3 | 76.8 | 76.9 | 77.2 | 73.2 |
| 3 U70263/Australia | 93.3 | 93.8 |  | 91.4 | 90.4 | 89.2 | 77.1 | 77.4 | 77.4 | 77.1 | 76.5 | 77.3 | 77.3 | 76.8 | 76.6 | 75.9 | 76.8 | 73.1 |
| 4 MT108680/USA | 93.4 | 94.2 | 94.6 |  | 90.1 | 88.7 | 77.4 | 77.6 | 77.6 | 77.9 | 76.6 | 77.2 | 77.2 | 77.3 | 77.1 | 76.3 | 77.1 | 73.3 |
| 5 KJ463422/USA | 93.8 | 93.8 | 94 | 94.2 |  | 89.9 | 77.5 | 77.7 | 77.6 | 77.7 | 77 | 77.3 | 77.2 | 77.5 | 76.9 | 76.9 | 76.8 | 73.3 |
| 6 AB897785/Japan | 94.1 | 94.6 | 94 | 94.2 | 94.2 |  | 77.9 | 77.9 | 77.8 | 77.8 | 77.1 | 77.6 | 77.6 | 77.2 | 76.8 | 76.7 | 77.4 | 73.5 |
| 7 MG649392/Italy | 86.5 | 86.6 | 86.3 | 86.8 | 87.4 | 87.2 |  | 93.2 | 93.3 | 86.3 | 77.2 | 77.4 | 77.4 | 77.5 | 76.9 | 76.8 | 77.9 | 73.7 |
| 8 MF102262/Switzerland | 86.2 | 86.4 | 86 | 86.4 | 87.3 | 86.9 | 95.8 |  | 99.3 | 86.1 | 77.4 | 78 | 78 | 77.9 | 77.1 | 77.2 | 77.6 | 73.8 |
| 9 MF102261/Switzerland | 86.2 | 86.5 | 86 | 86.4 | 87.3 | 86.8 | 95.9 | 99.2 |  | 86.2 | 77.3 | 77.9 | 77.9 | 77.9 | 77.2 | 77.3 | 77.7 | 73.8 |
| 10 MF102260/Switzerland | 86.1 | 86.4 | 86.2 | 86.9 | 87.1 | 86.8 | 92.1 | 92.1 | 92.1 |  | 77 | 77 | 77 | 77.2 | 76.8 | 77.2 | 77.8 | 73.8 |
| 11 AF144618/Germany | 85.9 | 86.1 | 85.7 | 86 | 86.5 | 86.4 | 86.1 | 86.3 | 86.4 | 86.6 |  | 76.9 | 76.9 | 77.3 | 77.1 | 76.6 | 76.9 | 73.4 |
| 12 MT648677/Germany | 85.6 | 85.8 | 85.7 | 86.2 | 86.8 | 86.5 | 87.3 | 87 | 87.1 | 87.3 | 87.3 |  | 99.8 | 80.4 | 80.2 | 77 | 77.7 | 73.4 |
| 13 KF925348/Germany | 85.6 | 85.8 | 85.7 | 86.1 | 86.7 | 86.4 | 87.3 | 87.1 | 87.1 | 87.1 | 87.2 | 99.7 |  | 80.4 | 80.1 | 77 | 77.6 | 73.3 |
| 14 KC963426/China | 85.5 | 85.8 | 85.4 | 86.5 | 86.6 | 86.4 | 86.5 | 86.4 | 86.5 | 86.9 | 86.4 | 90 | 89.9 |  | 87.2 | 77.4 | 77.4 | 73.2 |
| 15 HuLB14/China | 85.4 | 85.8 | 85.2 | 86.3 | 86.6 | 86.3 | 86.3 | 85.9 | 85.9 | 86.7 | 86.4 | 89.5 | 89.3 | 93.2 |  | 77 | 77.3 | 73.2 |
| 16 GU270877/Andorra | 85.5 | 85.6 | 85.2 | 85.6 | 85.8 | 85.8 | 86.5 | 86.5 | 86.6 | 86.5 | 86.4 | 86.7 | 86.8 | 87.1 | 86.6 |  | 79 | 73.6 |
| 17 KF918753/France | 85.7 | 86 | 85.3 | 85.9 | 86.3 | 85.8 | 87.2 | 87 | 87.1 | 87.7 | 87.1 | 87.4 | 87.3 | 86.8 | 86.6 | 88.3 |  | 74.1 |
| 18 LR824489/Italy | 81.4 | 81.8 | 81.2 | 82 | 82.4 | 82.3 | 81.8 | 81.7 | 81.8 | 82.1 | 81.8 | 82.5 | 82.4 | 82.1 | 82.2 | 81.9 | 82.8 |  |

TABLE S3. The viral strains used for phylogenetic and selection pressure analyses in the present study.

| **Virus species** | **GenBank accession number** | **Country** |
| --- | --- | --- |
| BDV | NC 003679 | Germany |
|  | MT108680 | USA |
|  | U70263 | Australia |
|  | AB897785 | Japan |
|  | MZ664275 | Netherlands |
|  | KJ463422 | USA |
|  | AF144618 | Germany |
|  | HuLB14 | China |
|  | KC963426 | China |
|  | KF925348 | Germany |
|  | MT648677 | Germany |
|  | MF102261 | Switzerland |
|  | MF102262 | Switzerland |
|  | MF102260 | Switzerland |
|  | MG649392 | Italy |
|  | GU270877 | Andorra |
|  | KF918753 | France |
|  | LR824489 | Italy |
| BVDV1 | KR866116 | China |
|  | MH490942 | China |
|  | MF693403 | China |
|  | MW250798 | United Kingdom |
|  | MW250799 | United Kingdom |
|  | MT977118 | Italy |
|  | MT977117 | Italy |
|  | MH490943 | China |
|  | KF896608 | Australia |
|  | MW250801 | United Kingdom |
|  | MW655627 | Switzerland |
|  | MW655631 | Switzerland |
|  | MW054939 | Italy |
|  | MW054934 | Italy |
|  | MW054936 | Italy |
|  | MW655632 | Switzerland |
|  | MW655625 | Switzerland |
|  | MW655629 | Switzerland |
|  | MW250803 | United Kingdom |
|  | MW250797 | United Kingdom |
|  | MW054936 | Italy |
|  | MW655630 | Switzerland |
|  | MW655628 | Switzerland |
|  | MW054935 | Italy |
|  | MH166806 | China |
|  | LC089876 | Japan |
|  | KC695810 | China |
| CSFV | KC503764 | India |
|  | KT119352 | China |
|  | KP233071 | China |
|  | AY259122 | Switzerland |
|  | AY775178 | China |
|  | MT799518 | China |
|  | MT799517 | China |
|  | MT799513 | China |
|  | MN399384 | South Korea |
|  | MN399383 | South Korea |
|  | MN399382 | South Korea |
|  | MN399380 | South Korea |
|  | MN558889 | South Korea |
|  | MN558888 | South Korea |
|  | MN558887 | South Korea |
|  | MN558886 | South Korea |
|  | MN558875 | South Korea |
|  | MN558874 | South Korea |
